# Systematic Review and Meta-Analysis on the Effects of Neurofeedback Training of Theta Activity on Working Memory and Episodic Memory in Healthy Population

**DOI:** 10.3390/ijerph191711037

**Published:** 2022-09-03

**Authors:** Wen-Hsiu Yeh, Ya-Ju Ju, Yu-Ting Liu, Ting-Yi Wang

**Affiliations:** 1 Institute of Basic Medical Science, National Cheng Kung University, Tainan 701, Taiwan; 2 Department of Physical Therapy, Shu-Zen Junior College of Medicine and Management, Kaohsiung City 821, Taiwan; 3 Institute of Allied Health Sciences, College of Medicine, National Cheng Kung University, Tainan 701, Taiwan; 4 Department of Medical Science Industries, Chang Jung Christian University, Tainan 711, Taiwan; 5 Department of Doctorate of Nursing Practice Program, University of Illinois, Chicago, IL 60612, USA

**Keywords:** cognition, memory, neurofeedback, randomized controlled trial, theta

## Abstract

The main purpose of this study was to investigate the effects of neurofeedback training (NFT) of theta activity on working memory (WM) and episodic memory (EM) in healthy participants via a systematic review and meta-analysis. A total of 337 articles obtained from electronic databases were assessed; however, only 11 articles met the criteria for meta-analysis after manually screening and eliminating unnecessary studies. A meta-analysis calculating the Hedges’ g effect size metric with 95% confidence intervals using random effects models was employed. Heterogeneity was estimated using I^2^ statistics. Theta NFT is effective in improving memory outcomes, including WM with a Hedges’ g of 0.56 [0.10; 1.02] (I^2^ = 62.9% and *p* = 0.02), and EM with a Hedges’ g of 0.62 [0.13; 1.10] (I^2^ = 42.04% and *p* = 0.01). Overall, the results suggest that theta NFT seems to be useful as nonpharmacological/adjunct training to improve WM and EM in healthy participants.

## 1. Introduction

An electroencephalogram (EEG) is a noninvasive method using multiple electrodes placed on the scalp to record the electrical activity of the brain. The rhythmic activity of the EEG is divided into several types of bands by frequency, i.e., delta, theta, alpha, beta, and gamma. Each frequency band has distinct cognitive functions. Theta activity (4–7 Hz) accompanies meditation, which is associated with the cortical inhibition of the sensory cortex and deep resting state without falling sleep [1,2]. The theta activity of the frontal midline has a positive correlation with cognition in healthy adults [3,4]. Pre-stimulus theta activity also predicts a role in correct source memory [5,6]. Moreover, event-related synchronization in theta activity reflects the cognitive process [1,7]. This evidence suggests that theta activity affects memory modulation in humans. The aim of this study was to investigate if positive memory function can be achieved by actively controlling theta activity.

EEG neurofeedback training (NFT), a model of biofeedback [8], is an operant conditioning paradigm where participants learn to influence the electrical activity of their brain. Participants need to wear a recording apparatus (i.e., an EEG cap) to record and display their brain activities as a feedback signal. NFT offers participants an opportunity to visualize their brain activities and then develop an efficient strategy to promote particular frequency activity within certain brain regions. EEG-NFT is a noninvasive and nonpharmacological approach and has been progressively advocated for psychological training since the 1990s [8]. To date, NFT has been shown to be effective in cognitive function in normal healthy individuals or neurological/psychiatric subjects. Numerous studies have indicated controversy concerning the therapeutic outcomes of available NFTs in certain populations, e.g., reading disability [9], traumatic brain injury [10], seizures [11], or post-traumatic stress disorder [12]. Neurological disorders with the occurrence of varied neural networks make it more difficult to determine the conclusive effects of NFT. To ascertain the efficacy of NFT on memory and to reduce the substantial heterogeneity in the selected population, a healthy population was selected for meta-analysis.

The effects of NFT of theta activity on cognitive functions and memory (e.g., working memory (WM) and episodic memory (EM)) were unknown in a recent study [13], and varied results have been obtained in previous studies [14,15]. Some studies indicated that theta NFT on memory enhancement has a significantly positive efficacy [13,16]. Several articles exhibited a minor memory improvement after theta NFT [17,18]. In contrast, some studies reported ineffective improvements in memory functions throughout theta NFT [18,19]. Further, a weak experimental design with no control group [20] or a small sample size [15] could generate controversial results from previous studies. Moreover, various training paradigms (i.e., different training durations and numbers of training sessions) are possible factors that contribute to the divergent results of theta NFT on memory [21]. A meta-analysis of theta NFT on memory was, essentially, performed to clarify the possible effect of theta NFT on memory.

We screened all previous studies that are related to the effect of NFT of theta activity on memory in healthy participants through a systematic review. We summarized the quality of each included study in terms of assessment of methodological quality, study bias (publication bias), participant characteristics (number and age), and training paradigm (electrode placement, feedback modality, and training frequency). Afterwards, we investigated the effect of NFT of theta activity on memory performance (WM and EM) through a meta-analysis of available previous works. This study provides qualitative and quantitative information to evaluate if theta NFT is a viable training approach for WM and EM in healthy populations.

## 2. Materials and Methods

This study followed Preferred Reporting Items for Systematic Reviews and Meta-Analysis (PRISMA) recommendations to undertake the search and analysis of the international scientific literature [22,23]. A registered number was recorded in the international prospective register of systematic reviews (PROSPERO No. CRD42021292660).

### 2.1. Data Sources and Searches

Three electronic bibliographic databases (PubMed, Embase, and the Cochrane Library) were used for the literature search. The search was conducted between 1 January 1990 and 30 June 2022. The PICOS framework was used to structure the search string as follows: P (population) = none; I (intervention) = theta neurofeedback; C (comparison) = no intervention, sham, or control group; O (outcome) = WM, EM, and cognition; and S (study design) = randomized controlled trial (RCT).

Two authors (W.-H.Y. and Y.-J.J.) searched the 3 electronic bibliographic databases for potential published articles. Search terms were as follows: (Theta OR theta) AND (neurofeedback OR Neurofeedback OR Electroencephalographic biofeedback OR electroencephalographic biofeedback OR EEG biofeedback OR EEG Biofeedback) AND (memor* OR Memor* OR cogniti* OR Cogniti*) AND (random group OR sham control OR sham group OR sham OR control OR control group OR non theta OR non theta group OR non-theta control).

### 2.2. Study Selection

We combined search results from different databases using EndNote reference manager software and deleted duplicate records. Two authors (W.-H.Y. and Y.-J.J.) independently searched and screened the titles, abstracts, and full texts identify potentially relevant studies. Then, relevant theta NFT studies were obtained and assessed to determine whether the articles met the inclusion criteria. Any disagreement was discussed and solved with the third author to reach a consensus on every relevant detail.

Studies were included if they met the following criteria:

(1) The experimental design was an RCT.
(2) The intervention was standard protocol EEG-NFT of theta activity, e.g., theta and alpha activities, theta/low beta ratio, or theta/beta ratio.
(3) The control group received active neurofeedback (e.g., randomly selected 4–6 Hz amplitude from the range of 10–25 Hz [14] or 2 Hz amplitude from the range of 10–24 Hz [24]), sham neurofeedback (e.g., simulated EEG activity from others [25]), or silent feedback (not receiving any stimulation or neurofeedback [13,19]).
(4) All the participants had no history of psychiatric or neurological disorders.
(5) Evaluation implicated the effect of theta activity on WM and/or EM. WM was defined as the structures and processes used for temporarily storing and manipulating information [26]. EM involved the ability to encode, consolidate, and retrieve past events along with their contextual details [27].

Studies were excluded if they were not full-text articles (i.e., abstracts, conference abstracts, e-posters, and posters), were not written in English, or did not present the standard deviation of WM to calculate the effect size.

### 2.3. Assessment of Study Quality 

Study quality was independently evaluated by two authors (W.-H.Y. and Y.-J.J.) using the PEDro scale [28,29]. This scale assesses the methodological quality of a study based on criteria such as randomized allocation, concealed allocation, baseline comparability, blind assessment (e.g., participants, therapists, assessors), adequate follow-up, intention-to-treat analysis, between-group comparisons, and point estimates and variability. One item on the PEDro scale (eligibility criteria) is related to external validity and is generally not used to calculate the method score, leaving a score range of 0–10. According to the PEDro scale, the studies were rated as excellent (9–10 points), good (6–8 points), fair (4–5 points), or poor (<4 points).

### 2.4. Data Extraction and Statistical Analysis

Data were independently extracted by two authors (W.-H.Y. and Y.-J.J.) using a standardized data extraction form. For all included studies, information was gathered based on the experimental design, participants’ sample size and age, EEG theta NFT characteristics (electrode positions, NFT modality, number of sessions, and duration of a session), and outcomes of interest (WM and EM).

Theta NFT on WM (e.g., n-back task or mental rotation tasks) and EM (e.g., word pair task) was collected as the outcome of interest. Several subgroup analyses including sample size, age, electrode placement, sensory feedback of NFT modality, number of training sessions, and duration of a session were performed. Data at the end of the intervention from the control and theta NFT groups were extracted. To facilitate standardized comparisons, the Hedges’ g effect size metric was adopted given that the measures used in the original studies were not on the same scale. Hedges’ g with 95% confidence intervals (CIs) of WM and EM in two groups for each study was used for calculation. Hedges’ random effects model for the pooled effect size (ES) was used in order to allow for variability among the participants and interventions, and thus to provide a more conservative ES estimate [30]. Hedges’ g effect sizes were interpreted as small (0.2), medium (0.5), and large (0.8). The I^2^ statistic reflecting a small (25%), medium (50%), or high (75%) degree of heterogeneity was used to quantify heterogeneity across studies [31]. A value of *p* < 0.1 was set for statistically significant heterogeneity. A forest plot was generated to show the Hedges’ g with the corresponding CIs for each study and the overall estimate of pooled random effects. A funnel plot and an Egger test were used to ascertain if publication bias was generated across studies. Review Manager 5.3 (Cochrane Collaboration, Copenhagen, Denmark) [28] and STATA 17 software were used for data analyses. Except for heterogeneity, *p*-values for all comparisons were two-tailed, and a value of *p* < 0.05 for all tests was considered statistically significant.

## 3. Results

### 3.1. Study Selection and Characteristics

**Figure 1:**
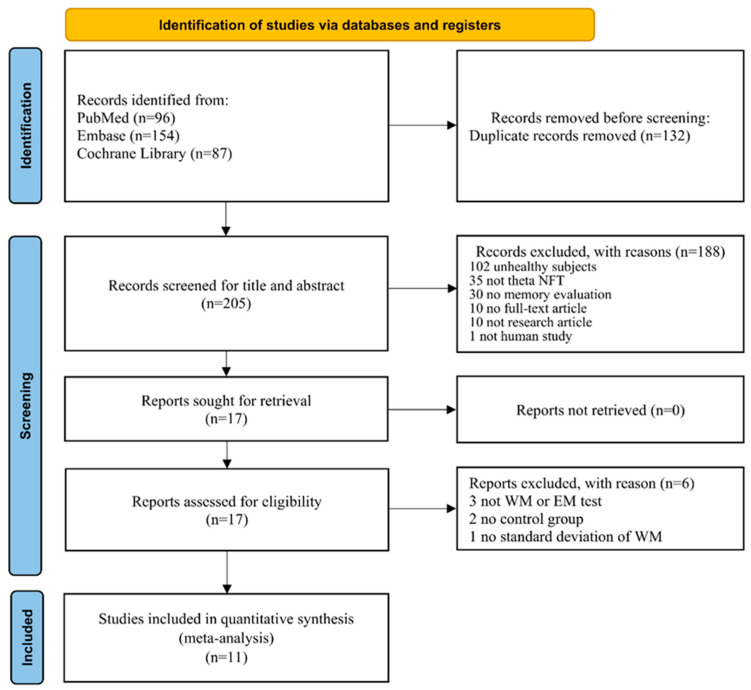
The Preferred Reporting Items for Systematic Reviews and Meta-Analyses (PRISMA) flowchart outlining the selection of studies included in the systematic review with meta-analysis.

Figure 1 shows the PRISMA 2020 schematic flow diagram for the process of study selection. A total of 337 titles and abstracts were initially identified, 132 were removed due to duplicates, and 205 titles and abstracts were screened for relevance. One hundred and eighty-eight studies were excluded due to the following reasons: inclusion of unhealthy participants (*n* = 102); theta NFT was not conducted (*n* = 35); absence of memory evaluation (*n* = 30); not a full-text article (e.g., conference abstract) (*n* = 10); not a research article (e.g., review paper) (*n* = 10); and not a human study (*n* = 1). Seventeen studies were sought for retrieval and were assessed for eligibility. Subsequently, six studies were excluded as three studies did not perform a WM or EM test, two studies lacked a control group, and one study did not present the standard deviation of WM. Finally, 11 studies were included for the qualitative and quantitative analyses.

Table 1 shows the characteristics of each study. The studies with an RCT were published from 2003 to 2021. Among the included studies, seven articles presented a two-arm RCT, two articles presented a three-arm RCT, and two articles presented a four-arm RCT. Overall, the studies included healthy participants, with an accumulated sample size of 328, ranging from 14 [25] to 50 [32]. The mean age of the overall population was ~34 years old.

Additionally, the studies varied in the EEG theta NFT, with differences in electrode location, feedback modality, training duration of a session, and number of training sessions. For the electrode location, eight studies (72.7%) primarily recorded frontal or fronto-central cortices, two studies (18.2%) recorded fronto-central-parietal or fronto-central-parietal-occipital cortices, and one study (9.1%) recorded fronto-parietal cortices. Of these studies, nine studies (81.8%) used NFT on theta activity, one study (9.1%) used NFT on theta and alpha activities, and one study (9.1%) used NFT on theta/low beta activity. For the sensory feedback of NFT, eight studies (72.7%) used visual feedback (VF), one study (9.1%) used audio feedback (AF), and two studies (18.2%) used audio-visual feedback (AVF). In the included studies, the number of training sessions ranged from 3 to 30 sessions, and the duration of a session ranged from 10 to 30 min.

### 3.2. Study Quality

Table 2 shows the authors’ judgment and the results of the study quality assessment by reporting the score of each subitem of the PEDro scale. The quality assessment performed according to the PEDro scale classified 7 of 11 studies (63.6%) as being of good quality, 3 of 11 studies (27.3%) as being of fair quality, and 1 of 11 studies (9.1%) as being of poor quality. Within the studies, among the subitems of the PEDro scale, 6 of 11 items (54.5%) ranged from 80% to 100% and, particularly, eligibility criteria, randomized allocation, concealed allocation, and baseline comparability were strongly meeting the criteria within the studies. Two of eleven items (18.2%) (blinded participants and intention-to-treat analysis) were meeting the criteria within the studies by 30% to 50%. Three of eleven items (27.3%) (blinded therapists, blinded assessors, and adequate follow-up) were meeting the criteria within the studies by 0% to 10%.

### 3.3. Risk of Bias across Studies

**Figure 2:**
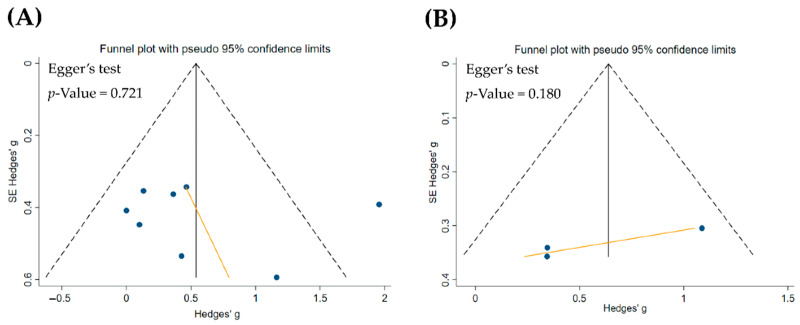
Funnel plot and Egger test assessing publication bias for theta activity neurofeedback training (NFT) on working memory (WM) (**A**) and episodic memory (EM) (**B**). Each point represents an independent study for the indicated associate.

Figure 2 shows no definite asymmetry in the forest plots. No significant publication bias existed statistically in the assessment based on the Egger test on studies for WM (*p* = 0.721) (Figure 2A) and EM (*p* = 0.18) (Figure 2B).

### 3.4. Synthesis of Results on Working Memory (WM)

**Figure 3:**
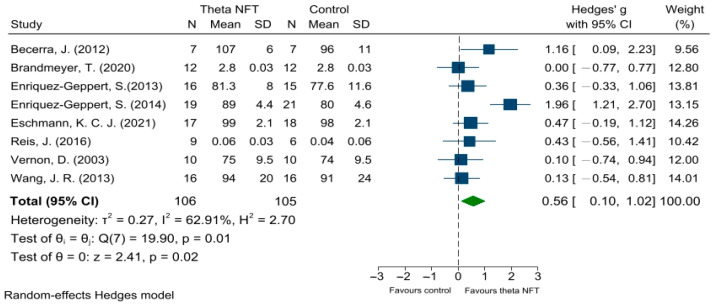
Forest plots showing meta-analysis of effect of NFT of theta activity on WM in healthy participants. CI = confidence interval; SD = standard deviation [14,18,19,24,25,33,34,35].

Figure 3 shows the effect of NFT of theta activity on the WM of healthy participants in 8 of 11 studies. Of these WM studies, three of eight studies (37.5%) exhibited significant WM enhancement compared with the control group. The results showed a significant overall effect (Hedges’ g = 0.56; *p* = 0.02; 95% CI = 0.10, 1.02; I^2^ = 62.9%), indicating that NFT of theta activity was beneficial for memory improvement compared with the control group.

Regarding the sample size, three of eight studies (37.5%) recruited ≤10 participants (7–10) into the theta group and ≤10 participants (6–10) into the control group. The results showed no significant overall effect (Hedges’ g = 0.50; *p* = 0.11; 95% CI = −0.11, 1.10; I^2^ = 17.4%). Five of eight studies (62.5%) recruited >10 participants (12–19) into the theta group and >10 participants (12–21) into the control group. The results showed no significant overall effect (Hedges’ g = 0.58; *p* = 0.10; 95% CI = −0.11, 1.27; I^2^ = 79.2%). We additionally analyzed studies that recruited ≤15 participants (16–19) into the theta group and ≤15 participants (15–21) into the control group. The results showed a tendency towards a significant overall effect (Hedges’ g = 0.72; *p* = 0.07; 95% CI = −0.09, 1.53; I^2^ = 81.9%). These results suggest that >15 participants or more would be a sufficient sample size for theta NFT on WM.

Regarding the age effect, six of eight studies (75%) recruited young adults (22–43 years old). The results showed no significant overall effect (Hedges’ g = 0.51; *p* = 0.09; 95% CI = −0.08, 1.09; I^2^ = 74.4%). Two of eight studies (25%) recruited elderly participants (>65 years old) to evaluate theta NFT on WM. The results showed a significant overall effect (Hedges’ g = 0.76; *p* = 0.04; 95% CI = 0.04, 1.49; I^2^ = 0%). These results suggest that theta NFT on memory showed a better improvement in older participants than in young adults.

We further considered the electrode placement. Six of eight studies (75%) placed electrodes over the frontal or fronto-central cortices. The results showed a significant overall effect (Hedges’ g = 0.58; *p* = 0.04; 95% CI = 0.03, 1.13; I^2^ = 69.1%). Two of eight studies (25%) placed electrodes over the fronto-centro-parietal or fronto-central-parietal-occipital cortices. The results showed no significant overall effect (Hedges’ g = 0.52; *p* = 0.37; 95% CI = −0.61, 1.65; I^2^ = 66.4%). These results suggest that theta NFT on the frontal or fronto-central cortices might affect WM.

Regarding the sensory feedback, six of eight studies (75%) used VF. The results showed a significant overall effect (Hedges’ g = 0.72; *p* = 0.01; 95% CI = 0.15, 1.28; I^2^ = 66.5%). One of eight studies (12.5%) used AF. The study reported that there was no significant WM improvement in the theta NFT group compared with the control group. One of eight studies (12.5%) used AVF. The results showed no significant WM difference between the theta NFT group and the control group. These results suggest that VF might be more effective for NFT on WM than AF and AVF.

Regarding the number of training sessions, all the studies (100%) performed theta NFT with >1 training session (7–30) and showed a significant overall effect (Hedges’ g = 0.56; *p* = 0.02; 95% CI = 0.10, 1.02; I^2^ = 62.9%). This result indicates that theta NFT with >1 training session would benefit WM performance.

Finally, the duration of a session was considered. A 20 min NFT presented a strong efficacy on memory in a previous study [31]. Two of eight studies (25%) designed a session of ≤20 min (15 min). The results showed no significant overall effect (Hedges’ g = 0.12; *p* = 0.66; 95% CI = −0.41, 0.65; I^2^ = 0%). Six of eight studies (75%) designed a session of >20 min (30 min). The results showed a significant overall effect (Hedges’ g = 0.72; *p* = 0.01; 95% CI = 0.15, 1.28; I^2^ = 66.5%). These results suggest that theta NFT of >20 min would improve WM. 

Table 3 shows a summary of the Hedges’ g effect sizes, 95% CIs, I^2^ statistics, and *p*-values of various outcome measures in the included WM studies.

### 3.5. Synthesis of Results on Episodic Memory (EM)

**Figure 4:**
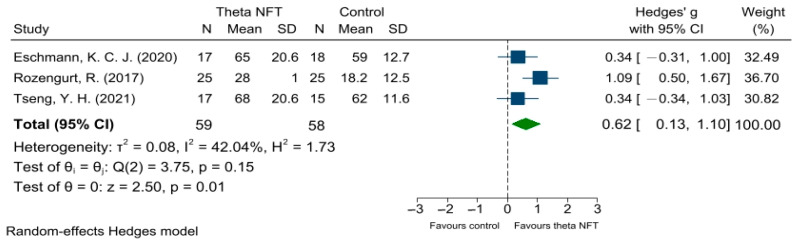
Forest plots showing meta-analysis of NFT of theta activity on EM in healthy participants. CI = confidence interval; SD = standard deviation [13,17,32].

Figure 4 shows NFT of theta activity on the EM of healthy participants in 3 of 11 studies. Of these EM studies, two of three studies (66.7%) exhibited a significant EM enhancement compared with the control group. The results showed a significant overall effect (Hedges’ g = 0.62; *p* = 0.01; 95% CI = 0.13, 1.10; I^2^ = 42%), suggesting that NFT of theta activity could improve EM performance compared with the control group.

Regarding the sample size, all the studies (100%) recruited >10 participants (17–25) into the theta group and >10 participants (15–25) into the control group. The results showed a significant overall effect (Hedges’ g = 0.62; *p* = 0.01; 95% CI = 0.13, 1.10; I^2^ = 42%). These results suggest that >10 participants might be a sufficient sample size for theta NFT on EM.

Regarding the age effect, all the studies (100%) recruited young adults (22–31 years old). The results showed a significant overall effect (Hedges’ g = 0.62; *p* = 0.01; 95% CI = 0.13, 1.10; I^2^ = 42%). These results suggest that theta NFT might be effective in young adults.

Further, we considered the electrode placement. Two of three studies (66.7%) placed electrodes over the frontal cortex. The results showed a significant overall effect (Hedges’ g = 0.74; *p* = 0.05; 95% CI = 0.01, 1.47; I^2^ = 61.9%). One of three studies (33.3%) placed electrodes over the fronto-parietal cortices. The study reported that there was no significant EM improvement in the theta NFT group compared with the control group. These results suggest that theta NFT on the frontal cortex might be more effective in EM.

Regarding the sensory feedback, two of three studies (66.7%) used VF. The results showed a significant overall effect (Hedges’ g = 0.73; p = 0.05; 95% CI = 0.00, 1.46; I^2^ = 63.7%). One of three studies (33.3%) used AVF. The results reported no significant EM difference in the theta NFT group compared with the control group. These results suggest that VF might be more effective for NFT on EM than AVF.

Regarding the influence of the amount of training sessions on the performance of theta activity on EM, all studies (100%) performed theta NFT with >1 training session (3–7). The results showed a significant overall effect (Hedges’ g = 0.62; *p* = 0.01; 95% CI = 0.13, 1.10; I^2^ = 42%), suggesting >3 training sessions would benefit EM performance. 

Finally, we considered the duration of a session. Two of three studies (66.7%) designed a session of >20 min (30 min). The results showed no significant overall effect (Hedges’ g = 0.34; *p* = 0.15; 95% CI = −0.13, 0.82; I^2^ = 0%). Only one study (33.3%) designed a session of ≤20 min (10 min). The study reported that there was a significant EM improvement in the theta group compared with the control group. These results suggest that a session of >10 min would benefit EM performance.

Table 4 shows a summary of the Hedges’ g effect sizes, 95% CIs, I^2^ statistics, and *p*-values of various outcome measures in the included EM studies.

## 4. Discussion

Eleven clinical trials were included in this study involving 328 participants (163 controls vs. 165 theta NFT participants). The results showed that some of the PEDro scale items, namely, eligibility criteria, randomization, allocation concealment, baseline comparability, and blinding of participants, were high-quality criteria. More than half of the included studies exhibited a good study quality. The blinding of therapists, blinding of assessors, and adequate follow-up items did not meet criteria in most of the included studies. The publication bias results exhibited a symmetrical funnel plot with no significant effect of theta NFT on WM and EM in the included studies. Moreover, theta NFT significantly improved WM and EM in healthy participants. Taken together, these results indicate a beneficial effect of NFT of theta activity on memory performance for healthy participants.

The small sample size in the treatment protocols of theta NFT in the included studies is believed to impact on outcomes. The results of the meta-analysis exhibited a small sample size (≤10), producing an insignificant overall effect of theta NFT on WM. Although a large sample size (>10) also resulted in an insignificant overall effect of theta NFT on WM, a sample size of ≥15 participants showed a tendency towards a significant overall effect. On the other hand, a large sample size (>10) resulted in a significant overall effect of theta NFT on EM. The difference in results suggests that the sample size may impact the effectiveness of NFT on WM and EM. A sample size of 15 participants or more is needed for the investigation of theta NFT on WM.

Age possibly affects the outcomes of NFT on memory. In a previous study, alpha NFT on memory improvement in young adults was more effective than in older participants [36]. The reason may be that the techniques of alpha NFT use sensory modalities such as vision for alpha and need a specific mental strategy to evoke alpha activity. For young adults, NFT with dual techniques was easily performed and had a high number of successful responders following a significant memory enhancement [37]. For elderly participants, aging was associated with a reduction in sensory modality stimuli and attentional load [38], and it was shown that elderly participants who underwent NFT with both visual stimuli and a mental strategy showed no significant change in the theta activity on memory improvement [35]. In contrast, only focused-attention meditation was shown to be an effective approach for regulating theta activity on memory [33]. Our meta results show that theta activity is beneficial for memory improvement in elderly participants. Moreover, NFT with an interface of visual modality for elderly participants may be a factor impacting performance.

NFT of theta activity in the frontal or fronto-central cortices exhibited a significant effect on WM and EM. Several studies demonstrated that the increase in theta activity in the frontal midline cortices is associated with a good cognitive performance [1,17,39]. An increase in theta activity, particularly in the parietal-occipital cortices, might be associated with a decrease in arousal [40]. Our meta-analysis revealed that theta NFT on the fronto-central-parietal or fronto-central-parietal-occipital cortices exhibited no significant effect on WM and EM. In the present study, a significant memory enhancement was shown from the range of the frontal to central cortices. These findings suggest that theta NFT has a greater effect in enhancing cognitive functions on the frontal midline cortices.

Previously, a study found that connectivity patterns of the frontal midline (FM) theta activity are directly related to the efficiency of WM maintenance and are affected by aging. Functional interactions of the FM cortex, which were proved to be facilitated during the retention period of the WM task, were also demonstrated to facilitate recognition memory. In the elderly, a substantially reduced connectivity strength of the FM region in association with decreased performance was evident [41]. These findings suggest that FM theta plays an important role in memory in the elderly. In the present study, the results of the meta-analysis also show that the effect of theta NFT of FM regions on WM in the elderly is better than in young adults.

NFT with the VF modality exhibited successful theta activity with WM and EM enhancement. Theta activity that is relatively modulated by the expected VF while having a plan in mind [42] is related to the promotion of a new information encoding and retrieval episode [43]. Our meta-analysis results found that VF benefitted theta activity in memory performance. In contrast, AF [19] or AF with visual stimulation [13,14] showed no significant difference in WM and EM of the theta NFT compared with those of the control group. Those phenomena suggest that VF may be a greater influence for theta NFT on memory than the others.

Regarding the intensity and dose of theta NFT, there was considerable variability among studies. The studies with a session duration of ≤20 min exhibited no significant overall effect of theta NFT on WM. In contrast, a duration of >20 min per session attained a significant level on WM. For EM, a duration of >20 min per session did not benefit memory improvement and exhibited an insignificant overall effect. Similar to a previous study [36], an NFT session longer than 20 min was shown to have an effect on WM. In this study, from the results of the meta-analysis, it was indicated that a training session of >20 min significantly enhanced the overall effect of theta NFT on WM. However, a training session longer than 20 min is recommended for theta NFT on EM.

Theta NFT with >1 session exhibited a significant effect on WM and EM. Moreover, theta NFT with only one session would affect WM enhancement. One study presented a significant WM enhancement throughout a session of NFT [16]. The results suggested an immediate effect on the WM process. Theta power enhancement has been associated with the encoding of new information [1,39], which is supported by a positive correlation of the theta amplitude with the load of new encoded information [43,44]. Moreover, the coding of a long period by theta activity is associated with episodic retrieval [45,46,47]. Of the included EM studies, in the present study, it was shown that 3–7 training sessions are effective for EM enhancement. However, theta NFT with only one session or >1 session would attain a positive effect on WM enhancement.

Numerous factors result in the efficacy of theta NFT on memory in healthy participants, including a sample size of >15, elderly participants >65 years old, a training strategy with just focused-attention meditation, and the region of cortices focusing on the frontal midline. Moreover, in the present study, it was indicated that >1 session with a training duration of >20 min could show a significant improvement in WM. For EM, an intensity and dose of 3–7 sessions would affect memory. However, a duration of over 20 min would be required. Compared with the NFT of alpha activity, a successful alpha NFT on memory needed a sample size of >10, alpha NFT on memory was more effective in healthy young adults than in the elderly, a training strategy with visual feedback and a mental strategy was beneficial for evoking the region of the parietal cortex [48], and the efficacy of alpha NFT on EM needed at least >10 training sessions with a duration of >20 min per session [36]. In a previous study conducted on fifteen heathy young adults, the results concerning alpha NFT suggested that positive effects were found for 12 sessions of NFT with a 25 min duration [49]. Taken together, the current study provides an insight into the successfulness of theta NFT on memory enhancement. Moreover, NFT of theta activity for memory performance seems be a more effective approach than that of alpha activity, particular in the elderly.

To the authors’ knowledge, this is the first systematic review and meta-analysis conducted exclusively on NFT of theta activity for WM and EM in a healthy population. The protocol for this review was pre-registered in PROSPERO, following PRISMA guidance for preparing a protocol, and reporting a systematic review and meta-analysis [50]. A comprehensive and rigorous review was undertaken in the current study. Therefore, the strengths of this study are represented by the appropriate selection criteria and study design as well as the credible results. This study highlights key points that could improve the WM and EM of healthy participants based on NFT of theta activity.

Nevertheless, some potential limitations exist in this study. Despite accurate search strategies, even if comprehensive and based on the PICOS framework, some potentially relevant studies of interest were possibly missed. Moreover, studies written in non-English languages were excluded in this study. Although one previous study suggested that excluding non-English publications from evidence syntheses did not change conclusions [51], English-language bias may affect the results of the evidence synthesis in the current study. Finally, the conclusions are based on the best available evidence synthesis, but it was not possible to avoid low-quality studies to minimize the risk of bias.

Caution is required when interpreting these findings given the several limitations in addition to the issues raised regarding the nature of the trials. Firstly, the critical appraisal of the included articles, blinding of therapists, and blinding of assessors presented a high risk of bias. Moreover, effect size estimates may be inflated owing to intention-to-treat analyses not meeting the criteria in most of the included studies. A rigorous methodology is important to improve the level of evidence of theta NFT on memory. An NFT checklist form would serve as a good guideline for trials [52]. Secondly, small samples of studies were taken into account in this meta-analysis, i.e., only three EM studies were included in this meta-analysis. Studies with a sample size of >10 are a more persuasive illustration of meta-analysis results according to a previous study [53]. Thus, more powered studies are needed in the future to investigate the impacts of NFT of theta activity on memory/cognition. Thirdly, baseline theta activity and spectral characteristics served as an important index for explaining the efficacy of theta on memory; however, they were incompletely demonstrated in most studies. Fourthly, intellectual properties including intelligence quotients, academic skills, and education levels of the participants served as an important index of determining the effectiveness of the training; however, they were insufficiently measured in the included trials. Finally, an extensive methodological revision (i.e., sensitivity analysis of key assumptions, diversification of assumptions, and replacement of weak assumptions with more realistic ones) is necessary to assess the actual effects of theta NFT before making assumptions.

## 5. Conclusions

We provided a complete and helpful guideline of theta NFT on WM and EM in a healthy population and analyzed all randomized controlled trials related to the efficacy of theta activity on both memories. The current study, which was a meta-analysis of available previous works, demonstrated significant WM and EM enhancement for healthy participants using NFT of theta activity in a large sample size (*n* = 328). Theta NFT could be added as a potential approach for facilitating memory power in cognitive trainings.

## Figures and Tables

**Table 1:** Characteristics of studies included in the meta-analysis.

First Author (Year)	Design	Sample		EEG-Theta NFT Characteristics			OOI	
		Participants	Age (Mean ± SD)	Electrode(s)	Modality (Feedback)	Sessions (min/Session)	WM	EM
Becerra, J. (2012) [25]	Two-arm RCT (Control vs. θ)	14 (7 vs. 7)	(67 ± 4.9 vs. 65.8 ± 2.4)	Fp1, Fp2, F7, F3, Fz, F4, F8, T3, C3, Cz, C4, T4, T5, P3, Pz, P4, T6, O1, O2	θ (VF)	30 (30)	WAIS-III	
Brandmeyer, T. (2020) [33]	Two-arm RCT (Control vs. θ)	24 (12 vs. 12)	(25.0 ± 3.0 vs. 25.0 ± 3.0)	Fpz, FZ, F7, F8, Cz, P7, P8, Oz	θ (VF)	8 (30)	N-back task	
Enriquez-Geppert, S. (2013) [34]	Two-arm RCT (Control vs. θ)	31 (15 vs. 16)	25 ± 3	Fz, FC1, FCz, FC2, Cz	θ (VF)	8 (30)	TBT	
Enriquez-Geppert, S. (2014) [18]	Two-arm RCT (Control vs. θ)	40 (21 vs. 19)	(25.8 ± 3.8 vs. 23.8 ± 2.7)	Fz, FC1, FC2, FCz, Cz	θ (VF)	8 (30)	TBT	
Eschmann, K. C. J. (2020) [17]	Two-arm trial (Control vs. θ)	36 (18 vs. 17)	(22.7 vs. 23.3)	Fz, P7	θ (VF)	7 (30)		Word pair
Eschmann, K. C. J. (2021) [24]	Two-arm trial (Control vs. θ)	36 (18 vs. 17)	(22.7 vs. 23.3)	Fz	θ (VF)	7 (30)	DMTS	
Reis, J. (2016) [35]	Four-arm RCT (Control vs. θ + α)	34 (6 vs. 9)	65.9 ± 6.6	FCz, Cz	θ+α (VF)	8 (30)	M. Rot.	
Rozengurt, R. (2017) [32]	Three-arm RCT (Control vs. θ)	75 (25 vs. 25)	(28.8 ± 6.5 vs. 30.9 ± 7.7)	Fz	θ (VF)	3 (10)		Graphic pair
Tseng, Y. H. (2021) [13]	Two-arm RCT (Control vs. θ)	32 (15 vs. 17)	21.6 ± 4.2	Fz	θ/low β (AVF)	3 (30)		Graphic pair
Vernon, D. (2003) [19]	Three-arm RCT (Control vs. θ)	30 (10 vs. 10)	22.1 ± 1.8	Cz	θ (AF)	8 (15)	SWMT	
Wang, J. R. (2013) [14]	Four-arm RCT (Control vs. θ)	32 (16 vs. 16)	(43.6 ± 2 vs. 43.4 ± 2.2)	Fz	θ (AVF)	12 (15)	MSRT	

Abbreviations: AVF, audio-visual feedback; CPT, continuous performance test; CST, conceptual span task; DMTS, delayed match to sample; M. Rot, matrix rotation task; MSRT, modified Sternberg recognition task; OOI, outcomes of interest; SMR, sensorimotor rhythm; SRT, spatial rotation task; SWMT, semantic working memory task; TBT, three-back task; VF, visual feedback; WAIS, Wechsler adult intelligence scale.

**Table 2:** Quality assessment of the studies included according to the PEDro scale.

Study	Criteria for the Quality Scoring											Score	Quality
	1	2	3	4	5	6	7	8	9	10	11		
Becerra, J. (2012) [25]	√	1	1	1					1	1	1	6/10	Good
Brandmeyer, T. (2020) [33]	√	1	1	1	1					1	1	6/10	Good
Enriquez-Geppert, S. (2013) [34]	√	1	1	1	1				1	1	1	7/10	Good
Enriquez-Geppert, S. (2014) [18]	√	1	1	1	1				1	1	1	7/10	Good
Eschmann, K. C. J. (2020) [17]	√		1	1						1	1	4/10	Fair
Eschmann, K. C. J. (2021) [24]	√		1	1						1	1	4/10	Fair
Reis, J. (2016) [35]	√	1								1	1	3/10	Poor
Rozengurt, R. (2017) [32]	√	1	1	1	1		1	1		1	1	8/10	Good
Tseng, Y. H. (2021) [13]	√	1	1	1	1					1	1	6/10	Good
Vernon, D. (2003) [19]		1		1						1	1	4/10	Fair
Wang, J. R. (2013) [14]	√	1	1	1	1				1	1	1	7/10	Good
Studies meeting criterion (%)	90.9%	81.8%	81.8%	90.9%	54.5%	0%	9.1%	9.1%	36.4%	100%	100%		

Notes: 1. Eligibility criteria; 2. Randomized allocation; 3. Concealed allocation; 4. Baseline comparability; 5. Blinded participants; 6. Blinded therapists; 7. Blinded assessors; 8. Adequate follow-up; 9. Intention-to-treat analysis; 10. Between-group comparisons; 11. Point estimates and variability. Check mark (√) indicates criterion evidenced.

**Table 3:** Summary of various outcome measures in the included WM studies.

Item	Outcome	Study	Hedges’ g	95% CI [LL, HL]	I^2^	*p*-Value
WM		[14,18,19,24,25,33,34,35]	0.56	[0.10, 1.02]	62.9%	0.02
Sample size						
	≤10 participants	[19,25,35]	0.50	[−0.11, 1.10]	17.4%	0.11
	>10 participants	[14,18,24,33,34]	0.58	[−0.11, 1.27]	79.2%	0.10
	≥15 participants	[14,18,24,34]	0.72	[−0.09, 1.53]	81.9%	0.07
Age effect						
	Young adults	[14,18,19,24,33,34]	0.51	[−0.08, 1.09]	74.4%	0.09
	Elderly (>65 years old)	[25,35]	0.76	[0.04, 1.49]	0%	0.04
Electrode placement						
	FC or FCC	[14,18,19,24,34,35]	0.58	[0.03, 1.13]	69.1%	0.04
	FCPC or FCPOC	[25,33]	0.52	[−0.61, 1.65]	66.4%	0.37
Sensory feedback						
	VF	[18,24,25,33,34,35]	0.72	[0.15, 1.28]	66.5%	0.01
Number of training sessions						
	>1 training sessions	[14,18,19,24,25,33,34,35]	0.56	[0.10, 1.02]	62.9%	0.02
Duration of a session						
	≤20 min	[14,19]	0.12	[−0.41, 0.65]	0%	0.66
	>20 min	[18,24,25,33,34,35]	0.72	[0.15, 1.28]	66.5%	0.01

Abbreviations: CI, confidence interval; FC, frontal cortex; FCC, fronto-central cortices; FCPC, fronto-centro-parietal cortices; FCPOC, fronto-central-parietal-occipital cortices; HL, high limit; LL, lower limit; VF, visual feedback.

**Table 4:** Summary of various outcome measures in the included EM studies.

Item	Outcome	Study	Hedges’ g	95% CI [LL, HL]	I^2^	*p*-Value
EM		[13,17,32]	0.62	[0.13, 1.10]	42%	0.01
Sample size						
	>10 participants	[13,17,32]	0.62	[0.13, 1.10]	42%	0.01
Age effect						
	Young adults	[13,17,32]	0.62	[0.13, 1.10]	42%	0.01
Electrode placement						
	FC	[13,32]	0.74	[0.01, 1.47]	61.9%	0.05
Sensory feedback						
	VF	[17,32]	0.73	[0.00, 1.46]	63.7%	0.05
Number of training sessions						
	>1 training sessions	[13,17,32]	0.62	[0.13, 1.10]	42%	0.01
Duration of a session						
	>20 min	[13,17]	0.34	[−0.13, 0.82]	0%	0.15

Abbreviations: CI, confidence interval; EM, episodic memory; FC, frontal cortex; HL, high limit; LL, lower limit; NFT, neurofeedback training; VF, visual feedback.

## Data Availability

The original contributions presented in the study are included in the article materials, further inquiries can be directed to the corresponding author.
